# Spine and dine: A key defensive trait promotes ecological success in spiny ants

**DOI:** 10.1002/ece3.6322

**Published:** 2020-04-29

**Authors:** Benjamin D. Blanchard, Akihiro Nakamura, Min Cao, Stephanie T. Chen, Corrie S. Moreau

**Affiliations:** ^1^ Committee on Evolutionary Biology University of Chicago Chicago IL USA; ^2^ Department of Science and Education Integrative Research Center Field Museum of Natural History Chicago IL USA; ^3^ CAS Key Laboratory of Tropical Forest Ecology Xishuangbanna Tropical Botanical Garden Chinese Academy of Sciences Menglun China; ^4^ Department of Statistics North Carolina State University Raleigh NC USA; ^5^ Departments of Entomology and Ecology and Evolutionary Biology Cornell University Ithaca NY USA

**Keywords:** competition, defense, morphological trait, predator–prey interactions, spines

## Abstract

A key focus of ecologists is explaining the origin and maintenance of morphological diversity and its association with ecological success. We investigate potential benefits and costs of a common and varied morphological trait, cuticular spines, for foraging behavior, interspecific competition, and predator–prey interactions in naturally co‐occurring spiny ants (Hymenoptera: Formicidae: *Polyrhachis*) in an experimental setting. We expect that a defensive trait like spines might be associated with more conspicuous foraging, a greater number of workers sent out to forage, and potentially increased competitive ability. Alternatively, consistent with the ecological trade‐off hypothesis, we expect that investment in spines for antipredator defense might be negatively correlated with these other ecological traits. We find little evidence for any costs to ecological traits, instead finding that species with longer spines either outperform or do not differ from species with shorter spines for all tested metrics, including resource discovery rate and foraging effort as well as competitive ability and antipredator defense. Spines appear to confer broad antipredator benefits and serve as a form of defense with undetectable costs to key ecological abilities like resource foraging and competitive ability, providing an explanation for both the ecological success of the study genus and the large number of evolutionary origins of this trait across all ants. This study also provides a rare quantitative empirical test of ecological effects related to a morphological trait in ants.

## Graphical abstract

We investigate potential benefits and costs of a common and varied morphological trait, cuticular spines, for foraging behavior, interspecific competition, and predator–prey interactions in naturally co‐occurring spiny ants (Hymenoptera: Formicidae: Polyrhachis) in an experimental setting. We find little evidence for any costs to ecological traits, instead finding that species with longer spines either outperform or do not differ from species with shorter spines for all tested metrics, including resource discovery rate and foraging effort as well as competitive ability and antipredator defense. These results suggest that spines promote broad ecological success in this hyperdiverse group of insects.

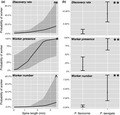

## 1 INTRODUCTION

Providing explanations for the origin and maintenance of morphological diversity and identifying subsequent consequences for ecological success are long‐standing goals of ecology. The evolution of particular morphological traits can determine outcomes of major ecological processes as wide‐ranging as establishment into novel niches (Azzurro et al., 2014), intra‐ and interspecific competition (Bennett, Riibak, Tamme, Lewis, & Pärtel, 2016), predator–prey interactions (Green & Côté, 2014), and mate choice (Roeder, Husak, Murphy, & Patten, 2019). This multitude of potential axes of selection within an environment that might influence morphological trait evolution and expression suggests that any given trait adapted for one ecological function may carry costs for, and trade‐off with, other functions (Kneitel & Chase, 2004; Stearns, 1989). Understanding the ecological benefits and costs of morphological traits requires quantitative assessments of trait function, and such assessments have been especially productive in plant systems (Agrawal & Fishbein, 2006; Mole, 1994; Tilman & Pacala, 1993), where experiments are generally easier than in animal systems. The depth of studies on functional traits in plants has allowed more complex theorizing about interacting suites of traits as opposed to, for example, univariate trade‐offs (Agrawal, 2007; Agrawal & Fishbein, 2006; Koricheva, Nykänen, & Gianoli, 2004), and highlights the efficacy of pursuing quantitative functional trait experimental methods to understand trait‐fitness relationships in diverse clades of interest.

**FIGURE 1:**
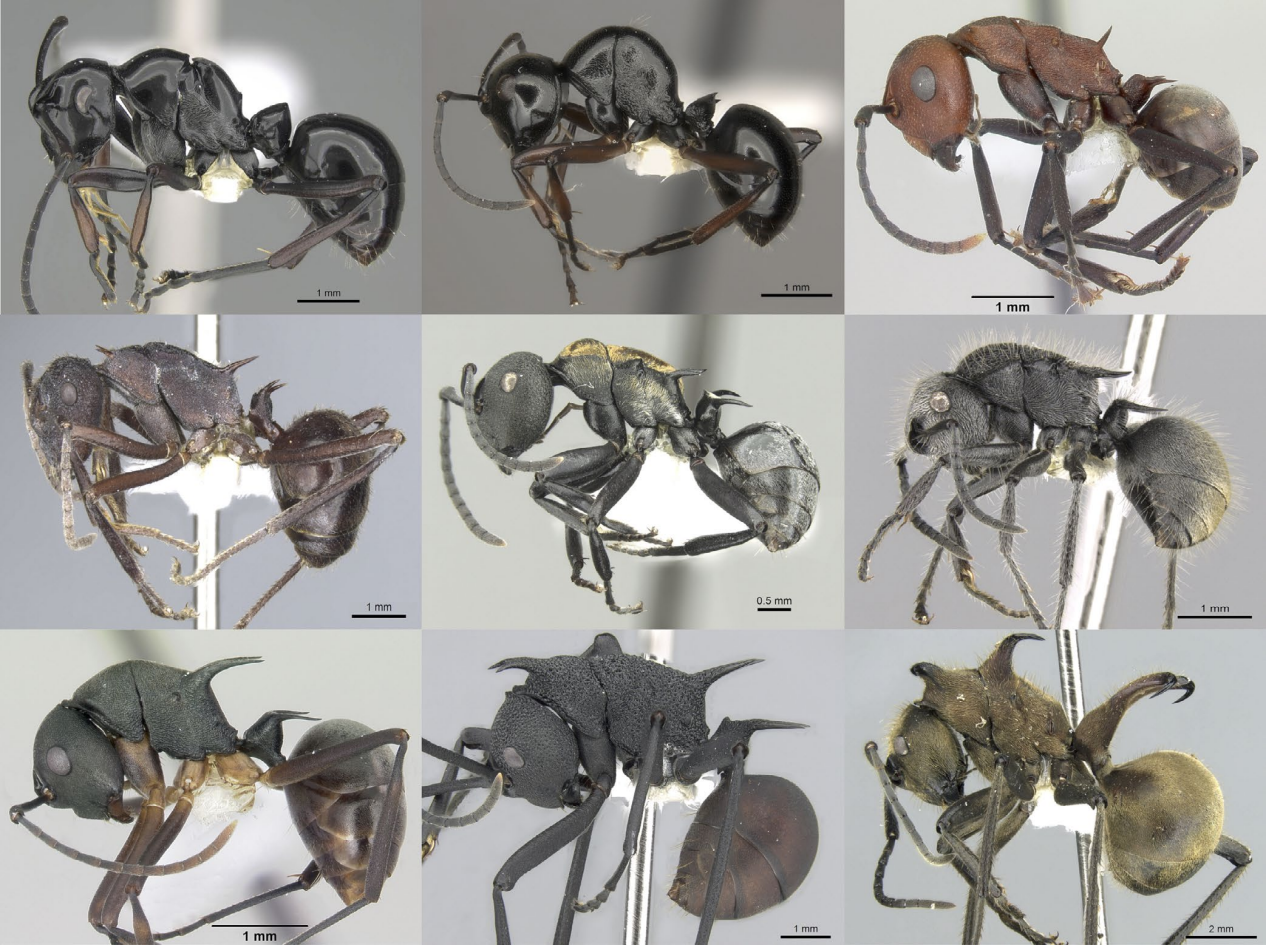
Morphological variation in the ant genus *Polyrhachis*. Species (and photo credit), from top left: *P. boltoni* (Michael Esposito), *P. robsoni* (Will Ericson), *P. deceptor* (April Noble), *P. loweryi *(Will Ericson), *P. ornata* (Michele Esposito), *P. lata* (Cerise Chen), *P. hippomanes* (April Noble), *P. armata* (Estella Ortega), *P. ypsilon* (Estella Ortega). From www.antweb.org under a Creative Commons Attribution License. Accessed 13 June 2019

Here, we investigate the role of a common morphological trait, cuticular spines (“spinescence”), on foraging behavior, interspecific competition, and predator–prey dynamics in *Polyrhachis* ants in a laboratory setting. Spines vary remarkably across ant species, sometimes exhibiting an extreme range of phenotypes within a single genus, from no spines to multiple large and curved thorn‐like projections reaching lengths matching the length of the entire thorax (Figure 1; Sarnat, Fischer, & Economo, 2016). Importantly, ant spines are typically greatly reduced or absent in the reproductive caste (queens and males), and thus, their function likely differs from well‐studied cases of spines undergoing sexual selection (e.g., Emlen, 1997). Recent work shows that spinescence is associated with elevated species diversification rates across all ants (Blanchard & Moreau, 2017). Furthermore, Ito, Taniguchi, and Billen (2016) demonstrated that spines confer defense against a vertebrate predator, the Japanese tree frog *Hyla japonica*, showing that the survival rate of workers significantly decreased when spines of the spiny ant species *Polyrhachis lamellidens* were experimentally removed. Pekár, Petráková, Bulbert, Whiting, and Herberstein (2017) find decreases in predation from both vertebrate and invertebrate predators in a study that bins all defensive traits—including spines—into a single trait, but Mikolajewski, Johansson, Wohlfahrt, and Stoks (2006) demonstrate that spines in dragonfly larva are ineffective in defending against invertebrate predators. These results suggest that a greater ecological understanding of spine function and impact on fitness would significantly contribute to our understanding of drivers of ecological success, morphological divergence, and species diversity in ants, a globally dominant group of insects.

While experimental tests of morphological trait function are rare in ant research (e.g., Ito et al., 2016; Larabee & Suarez, 2015; Poulsen, Bot, Currie, & Boomsma, 2002), existing work suggests some specific associations between traits and ecological functions, for example, between head, eye, limb, and total body size and diet, foraging strata, and guild (Gibb et al., 2015; Silva & Brandão, 2010; Weiser & Kaspari, 2006). Utilizing laboratory experiments, Larabee and Suarez (2015) found fitness increases from high‐powered mandibles that allow trap‐jaw ant workers to propel themselves away from antlion attacks. Fieldwork and laboratory work on tropical canopy ants suggest species assembly is mediated, at least in part, by body size variation (Fayle, Eggleton, Manica, Yusah, & Foster, 2015), while Retana, Aman, and Cerdá (2015) showed that several morphological traits (e.g., worker polymorphism) were associated with foraging strategy, behavioral dominance, and other ecological traits. Given such known associations between morphological and ecological traits in ants, it is likely that many traits carry benefits and costs within‐ and between‐ant species.

We focus on competition and predation as two selection pressures likely to drive adaptation in ants. Numerous studies have used field and laboratory methods to better understand ant competition. One common approach is to address a putative trade‐off between resource discovery and resource dominance as different strategies used to minimize competitive interactions between ant species (Bertelsmeier, Avril, Blight, Jourdan, & Courchamp, 2015; Davidson, 1998; LeBrun & Feener, 2007; Parr & Gibb, 2012). Field studies have targeted the role of dominance hierarchies in structuring communities (Savolainen & Vepsäläinen, 1988; Stuble et al., 2013), as well as the mechanisms involved in competitive exclusion by native ants (McGlynn & Parra, 2016) and invasive ants (Holway, 1999). Laboratory studies have provided support for theorized species assembly rules in tropical canopy ants (Fayle et al., 2015) and the influence of intraspecific variation in competitive dynamics (Lichtenstein, Pruitt, & Modlmeier, 2015; Thomas, Tsutsui, & Holway, 2004).

Relative to competition, the influence of predation on ant ecology and evolution has been largely underexplored (Cerdá, Xavier, & Retana, 2013). The studies that do exist suggest predator–prey dynamics are a promising area of research, providing explanations for important behaviors like nest relocation (McGlynn, Carr, Carson, & Buma, 2004) and significant variation in morphological traits like mandibular shape (Larabee & Suarez, 2015) and cuticular spines (Ito et al., 2016). Understanding compelling putative top‐down mechanisms of diversification, like that proposed by the escape‐and‐radiate hypothesis (Ehrlich & Raven, 1964), requires experimental work. Therefore, extant studies in ants and other systems should motivate a greater focus on the effects of predators on ecological interactions and species diversification.

In this study, we test for benefits and costs of spines through a novel investigation that directly connects a morphological trait to multiple ecological trait outcomes in this insect group. Under an expectation that spines are adapted to repel predators (Dornhaus & Powell, 2010), we predict that spines are positively associated with antipredator defense. Furthermore, we predict that the associated release from predator pressure allows workers with spines to exhibit increased, conspicuous resource foraging abilities and potentially enhanced abilities in other ecological traits like competitive ability as well. This second prediction can be explained through two plausible mechanisms. First, if predation exerts significant selection for predator avoidance behaviors that decrease foraging efficiency by promoting inconspicuous foraging strategies such as foraging in smaller numbers or using circuitous foraging routes, then we predict that selection for antipredator defensive spines in a given population will promote conspicuous foraging traits like persistent occupancy of a foraging area and a higher number of foraging workers. Second, if such a population no longer experiences significant energetic loss from predation, a colony may be able to invest that energy into other key traits like competitive ability (e.g., aggression) and thus invest more in such traits relative to competitors that do not benefit from spine defenses.

However, despite the expected mechanisms described above, various potential costs of spine production may alternatively drive a negative association between antipredator defense and other ecological traits including resource foraging and competitive abilities. The energy expended to produce spines, which in addition to cuticle can also contain muscle tissue (Sarnat, Friedman, Fischer, Lecroq‐Bennet, & Economo, 2017), may trade off with energetic investment elsewhere (e.g., number of workers produced or muscle production in the legs). Particularly in the more extreme trait states, spines may reduce maneuverability for workers in their environment and in competitive interactions. Wilson (1959) posited that spines reduce the number of nesting spaces available to a species, as the more confined spaces in twigs and subterranean habitats may preclude occupancy by workers bearing such rigid, protruding structures. This constraint on movement might explain a negative association between spines and foraging abilities (such as resource discovery rate) or ability in competitive interactions. Furthermore, we might expect that ant species investing in defense invest less in competitive ability, a trade‐off found in many other taxa (cotton plants: Karban, Brody, & Schnathorst, 1989; flies: Kraaijeveld & Godfray, 1997; crustaceans: Wellborn, 2002; algae: Yoshida, Hairston, & Ellner, 2004; mosquitofish: Langerhans, 2009; salamanders: Urban & Richardson, 2015). Notably, such costs may exert selection against spines in a population, or a clade of species, on a different timescale than benefits exerting selection for spines, and temporal differences in selection may explain the repeated gains and losses of spines across the ant tree of life (Blanchard & Moreau, 2017).

Thus, to evaluate the potential ecological benefits and costs of spines, we ask the following: (a) Do species with spines invest more (or less) time and effort foraging for resources? (b) Is there a positive (or negative) relationship between spines and competitive ability? (c) Do species investing in spinescence have higher (or lower) survival under predator–prey conditions? Our work provides a quantitative empirical test of performance related to a morphological trait in ants as well as an assessment of morphological trait‐mediated ecological trade‐offs and potential trait‐based drivers of ecological success in a diverse insect group.

## 2 MATERIALS AND METHODS

We worked at the Xishuangbanna Tropical Botanical Garden (XTBG) in southern Yunnan province in southern China. XTBG and the surrounding regions in Xishuangbanna experience a tropical climate and are part of the Indo‐Burma biodiversity hot spot (Myers, Mittermeier, Mittermeier, Da Fonseca, & Kent, 2000). Correspondingly, Yunnan is home to the richest ant fauna in China with over 450 documented species (AntMaps.org; accessed February 2019; Janicki, Narula, Ziegler, Guénard, & Economo, 2016). One of the most speciose and morphologically diverse ant genera in this region is the spiny ant genus *Polyrhachis* Fr. Smith, with around 30 species currently known from the province (AntMaps.org). Most *Polyrhachis* species contain some number of exoskeletal spines up to one pair each at three locations along the mesosoma with an additional pair sometimes located on the petiole, although some species have no spines (Figure 1). All species exhibit worker monomorphism. XTBG is well‐suited for this work as numerous *Polyrhachis* species exhibiting a range of spinescence overlap in microhabitat at this location (Guénard & Dunn, 2012), and several species often nest in close proximity and share similar nesting behavior, using larval silk to construct nest structures between or on the underside of leaves (Robson & Kohout, 2007). Furthermore, most if not all *Polyrhachis* species have a diet low on the trophic scale that relies on Hemipteran mutualists, plant exudes, and other opportunistically acquired sugary resources in the environment (Liefke, Dorow, Hölldobler, & Maschwitz, 1998; Staab, Fornoff, Klein, & Blüthgen, 2017). This overlap in geographic location, microhabitat, and niche makes it likely that these species regularly interact in their environment, experiencing shared selection pressures from interspecific competition and predation.

During June and July of 2017 and 2018, we collected *Polyrhachis* colonies in and around XTBG. We kept the colonies in the field station laboratory in plastic containers (17 × 11.5 × 10 cm), and regularly provided each colony a standard liquid sugar diet (50% sugar/50% water solution) as a rough approximation of their natural sugary food sources. Species were identified in the laboratory using the primary literature (Kohout, 2010, 2014; Xu, 2002a, 2002b) and images from AntWeb.org (accessed June–July 2017). Species voucher specimens were deposited in the Field Museum of Natural History in Chicago, IL, USA, and the Southwest Forestry University Specimen Hall in Kunming, Yunnan, China.

### 2.1 Resource discovery rate, foraging effort, and competitive ability trials

We first conducted resource discovery trials with 89 colonies, representing 11 *Polyrhachis* species (Table S1). We explored the effect of spinescence on time and worker investment by testing associations between spines and resource discovery rate and foraging effort. Approximately 48 hr before a trial for a given colony, we removed the food from the colony's container. At the start of the trial, we removed the lid from the colony's container and placed it into a larger bin filled with 2–3 cm of water to create a moat (preventing escape) and let it sit for 5 min for colony acclimation. We then set a smaller container, with food (50% sugar/50% water solution), into a second larger bin filled with water, and used standardized manufactured wood pieces (chopsticks) as a bridge to connect this food chamber to the colony container (Figure S1). Following attachment, we recorded the number of ants present in the food container every minute for 60 min. We noted the time until the first ant entered the food chamber (“Discovery Rate”), and two metrics of foraging effort: colony presence/absence in each one‐minute time bin with “present” indicating at least one ant present in the food chamber (“Worker Presence”), and number of workers present in the food chamber during each one‐minute time bin (“Worker Number”). We also documented species identity, colony size (counted manually following all trials), body size (determined for a species based on the diagonal length of the mesosoma, Weber's length, for one representative worker), and spine length (determined for a species based on the combined length of spines across one half of the mesosoma and petiole for one representative worker; Table S1). While our metric for spine length is a combined variable that does not necessarily capture the full variation of spine morphologies in the genus, we believe it is a good first approximation of overall spinescence.

We then conducted multispecies interspecific competition trials, which served as a broad test of spine‐mediated competitive ability in *Polyrhachis*. These included 72 colonies from 11 different species (Table S1), with pairings between colonies randomized except for an attempt to maintain similar estimated colony sizes and collection date between species pairs. Our experimental design and trial methods matched the resource discovery trials, except paired colonies were attached to the same food container. We documented the number of ants present in the central chamber and in the opposing colony's container every minute for 60 min, for both colonies in a given trial. We summed the proportion of the colony present in the central chamber and in the opposing colony's container, averaged over the trial period and across trials within a species, and used this value to estimate competitive ability (see “Statistical tests”, below).

**FIGURE 2:**
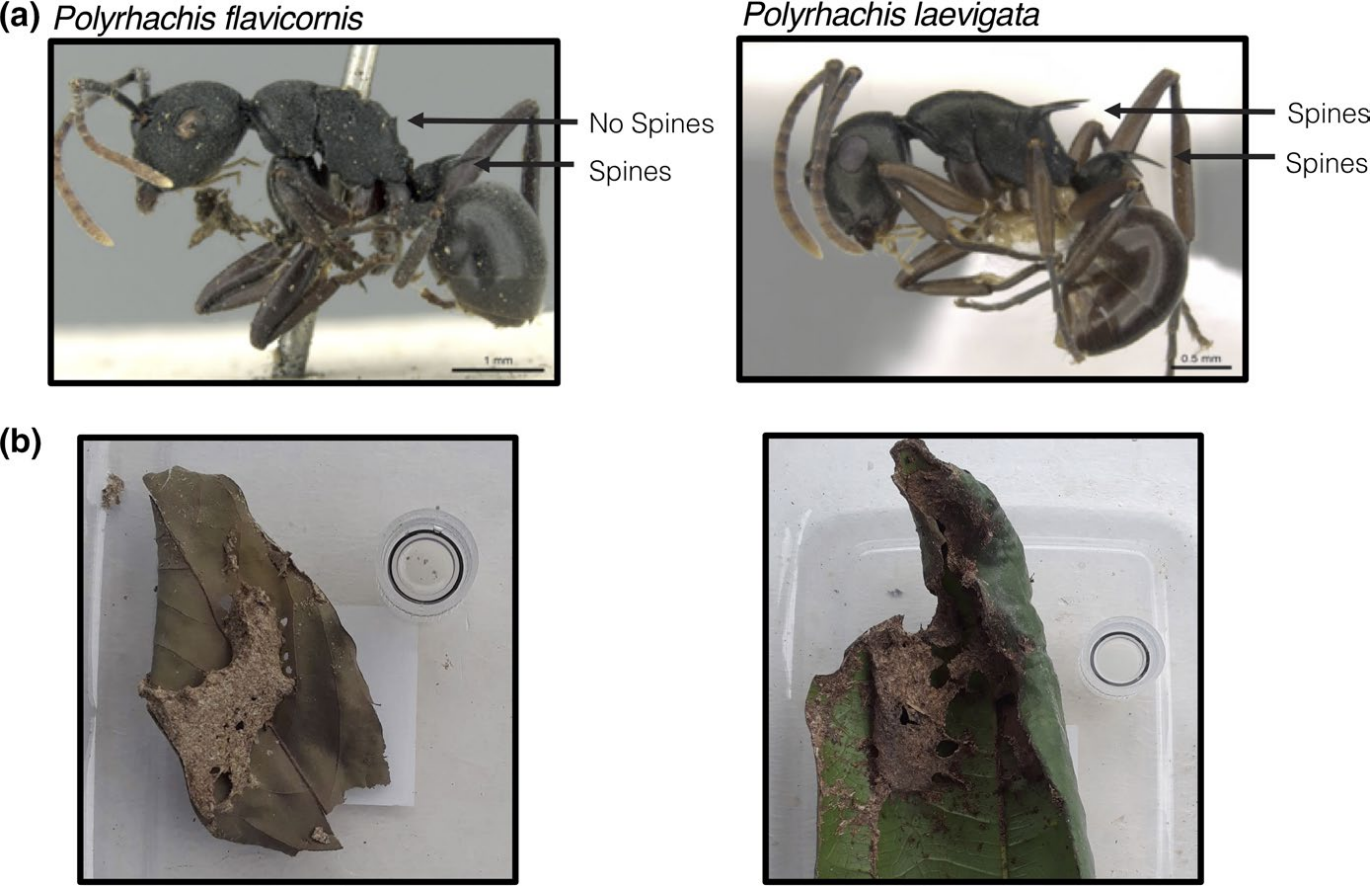
Comparison between *P. flavicornis* and *P. laevigata* (a) morphology, and (b) nests (after collection). Image credit: (a) Estella Ortega and Michele Esposito, from www.antweb.org under a Creative Commons Attribution License. Accessed 13 June 2019. (b) B.D. Blanchard

Although a multispecies approach is ideal for identifying effects from spines distinct from other species effects, our multispecies trials suffer from low sample sizes for several species and thus limited statistical robustness overall. Therefore, we also utilized a two‐species approach. From the multispecies trials, we identified two focal species: *Polyrhachis flavicornis*, with two short petiolar spines, and *P. laevigata*, with four medium‐length spines on the propodeum and petiole (Figure 2a). These species are naturally co‐occurring and have very similar body size, overall appearance, arboreal nest architecture, and geographic ranges (Figure 2b; AntMaps.org). Colonies of both species are monogynous (single queen) and monodomous (single nest dome), unlike at least two species in the multispecies trials, minimizing potential associated confounding factors. Furthermore, we could collect a relatively large number of individual colonies of each species, as they are both common in our collection area. Therefore, *P. flavicornis* and *P. laevigata* were well‐suited for testing our hypotheses while minimizing the likelihood of various other confounding factors, although we acknowledge that two‐species tests remain limited in their utility to robustly assess adaptive hypotheses (Garland & Adolph, 1994).

The two focal species, *P. flavicornis* and *P. laevigata*, were used for additional trials using the same experimental design and methods as for the multispecies discovery trials. The two‐species trials included 30 colonies from each species, for a total of 60 colonies (Table S2). As with the multispecies trials, “Discovery Rate,” “Worker Presence,” and “Worker Number” data were collected over 60‐min trials. Furthermore, to test the impact of spines on interspecific competition and the potential for an ecological trade‐off between antipredator defense and competitive ability, we conducted competition trials as we did for the multispecies dataset, pairing colonies to minimize differences in estimated colony size and collection date (*n* = 30 pairs). Through comparing the resource discovery rate and foraging effort results when colonies were paired versus when they were alone, we could assess shifts in abilities, where a significant increase or decrease in outcomes for one species (but not the other) would signal superior or inferior competitive ability, respectively.

### 2.2 Antipredator defense trials

To assess the function of spines in repelling invertebrate predators, in August 2018 we hand collected adult individuals of *Siler semiglaucus*, a common and widespread ant‐specialist jumping spider that preys on adult ants (Jackson & Olphen, 1992; World Spider Catalog, accessed August 2019), at XTBG. Although Mikolajewski et al. (2006) found that spines were ineffective against invertebrate predators of aquatic dragonfly larvae, our terrestrial system is significantly different; furthermore, ant spines may present a physical barrier that limits the ability of jumping spiders to access the main body cavity at vulnerable joints along the body. We collected individuals of *S. semiglaucus* by beating shrubs over an upturned umbrella and placed each spider in individual plastic tubes (10 × 4 cm) with a single flower petal to maintain humidity. After approximately three days (to ensure interest in prey), we placed a single spider into a small plastic box (10 × 10 × 6 cm) with an ant worker from one of the two focal species (*P. laevigata* and *P. flavicornis*; *n* = 11 and *n* = 12, respectively) and assessed ant survival after 1 and 24 hr. As controls, we placed 10 ants of each species in individual boxes (1 ant/box) without spiders present.

### 2.3 Statistical tests

For the multispecies resource discovery rate and foraging effort trials and the two‐species resource discovery rate, foraging effort, and competition trials, we utilized generalized linear models (GLMs) and generalized linear mixed models (GLMMs) using the “glm” and “glmer” functions in R packages “stats” and “lme4” (Bates, Maechler, Bolker, & Walker, 2015; R Core Team, 2018). For the multispecies trials, we modeled Discovery Rate, Worker Presence, and Worker Number using GLMMs. We assumed Discovery Rate followed a geometric distribution, which measures the number of intervals (time bins) before the first “success” (i.e., appearance of the first worker in the food chamber). We assumed Worker Number per time bin followed a binomial distribution, which measures the number of successes (workers in the food chamber) out of the total colony size. Similarly, we assumed the response for Worker Presence, a binary trait where “0” represents the absence of any workers and “1” represents the presence of at least one worker, followed a Bernoulli distribution. For all GLMMs, spine length and body size were modeled as fixed effects, while species identity was treated as a random effect. For Discovery Rate and Worker Presence, colony size was also included as a fixed effect. For Worker Presence and Worker Number, time bin was added as a fixed effect, as we expect progression of time during the trial to be important and want to account for this effect. We also treat specific colony identity as an additional random effect.

We conducted similar analyses for our two‐species resource discovery rate, foraging effort, and competitive ability trials. As we were interested in comparing *P. flavicornis* (short spines) and *P. laevigata* (medium spines), we modeled species as a fixed effect rather than random effect. As a result, the model for Discovery Rate was a GLM rather than a GLMM. Furthermore, to assess behavioral differences resulting from competition, results from discovery rate and foraging effort (alone) trials were compared to results from competitive ability (paired) trials for the same species for each of the two species, using the same GLMM/GLM framework for Discovery Rate, Worker Presence, and Worker Number.

For all GLM/GLMM analyses, we confirmed, using Pearson's correlation coefficient in the base R function “cor”, that all variables included in each model exhibited a correlation coefficient < 0.8 (Table S3).

For the multispecies test of competition, we used the Colley matrix (Colley, 2002). The Colley matrix, which was originally designed for ranking football teams but does not carry any sports‐specific assumptions, is well‐suited to situations with a large number of competitors but a very small number of pairings out of the total possible number of pairwise competitive events (LeBrun & Feener, 2007; Stuble et al., 2013). This method uses “win‐loss” data and incorporates the relative strength of each competitor in ranking all competitors according to interaction outcomes. We considered each species as a competitor, and a “winner” for any given pairing to be the colony that had a higher percentage of the colony present in the containers averaged over the trial period. We then conducted a phylogenetic linear regression between the resulting Colley matrix metric values and relative spine length (spine length divided by body size), using the “phylolm” function in the R package “phylolm” (Ho & Ané, 2014). We utilize a dated molecular phylogeny of the genus, inferred using a genome‐wide sequencing approach, that includes all taxa in this study (Blanchard and Moreau *in prep*).

To evaluate differences in ant prey survival, we used a chi‐square test to compare differences between species after 1 hr and after 24 hr, with each box treated as an independent observation.

## 3 RESULTS

### 3.1 Resource discovery rate and foraging effort

**FIGURE 3:**
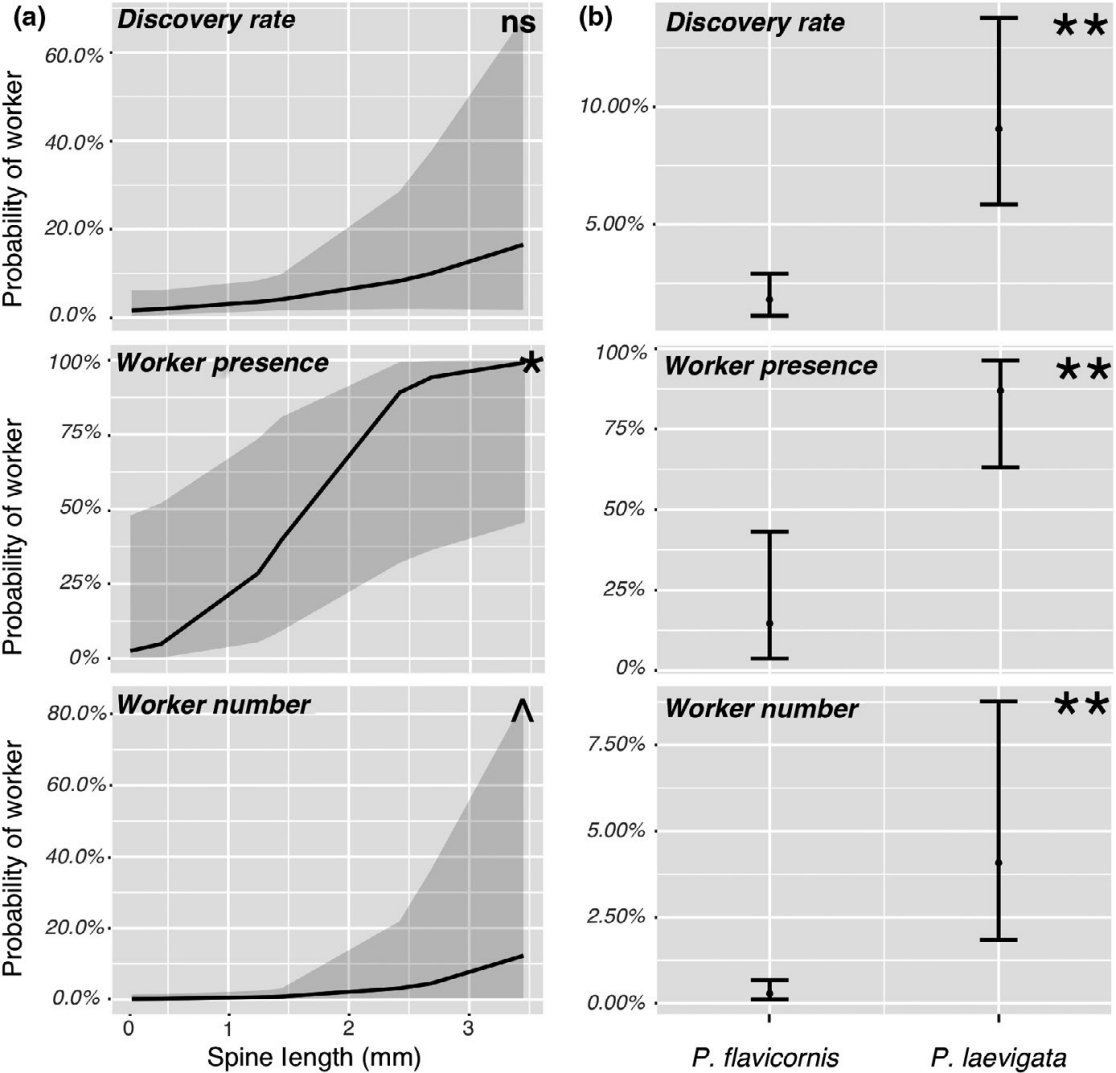
Resource discovery rate and foraging effort for (a) multispecies (*n* = 9, *Cyrtomyrma* excluded) trials and (b) two‐species trials. Grey zones and bar lines indicate 95% prediction interval boundaries, that is, the interval expected to contain 95% of future observations for a given spine length or species, respectively. ^*p* < .1, *p < .05, **p < .001, ns, not significant (*p* > .1)

For our trials across multiple species (*n* = 9; Table S4), Discovery Rate was not significantly associated with spine length, although there was a trend suggesting a positive relationship (*Z* = 1.51, *p* = .13; Figure 3a). Discovery Rate was not associated with body size but was positively associated with colony size (*Z* = 0.06, *p* = .95; and *Z* = 2.54, *p* = .01, respectively), suggesting faster discovery rates for species with larger colony sizes. Worker Presence (i.e., colony presence/absence in each one‐minute time bin with “present” indicating at least one ant present in the food chamber) was significantly positively associated with spine length, and Worker Number was somewhat significantly positively associated with spine length (*Z* = 2.165, *p* = .03; and *Z* = 1.74, *p* = .08, respectively; Figure 3a), while neither were associated with body size (*Z* = −0.38, *p* = .70; and *Z* = −0.26, *p* = .80, respectively). Colony size was not associated with Worker Presence (*Z* = 1.58, *p* = .12), and both Worker Presence and Worker Number were positively associated with time during the trial (*Z* = 8.19, *p* < .01; and *Z* = 24.59, *p* < .01, respectively).

We observed that the two species in the *P. (Cyrtomyrma)* subgenus, representing eight (9%) of the 89 colonies in our dataset, fell far outside the general trends across all 11 species. Furthermore, these species exhibit a distinct, extreme defensive behavior of rapidly dropping off leaves and onto the ground when even moderately disturbed by a vertebrate (pers. obs.). Given these observations, and our focus on morphological defenses as opposed to any alternative defenses, we excluded these eight colonies from our main results (reported above) but include them in Figure S2 and Table S4. Notably, the overall trends were similar, although less statistically supported, even when including the eight colonies from these two species exhibiting distinct, nonmorphological defensive behaviors.

In the two‐species trials (Table S4), *P. laevigata*, the species with longer spines, had a significantly faster Discovery Rate and higher degree of Worker Presence and Worker Number compared to *P. flavicornis* (*Z* > 3.48, *p* < .01 in all cases; Figure 3b). Colony size did not impact Discovery Rate (*Z* = 0.20, *p* = .84) but was positively associated with Worker Presence (*Z* = 2.40, *p* = .02) in the two‐species case. Time was positively associated with both Worker Presence and Worker Number (*Z* > 15.73, *p* < .01 for both).

### 3.2 Competitive ability

**FIGURE 4:**
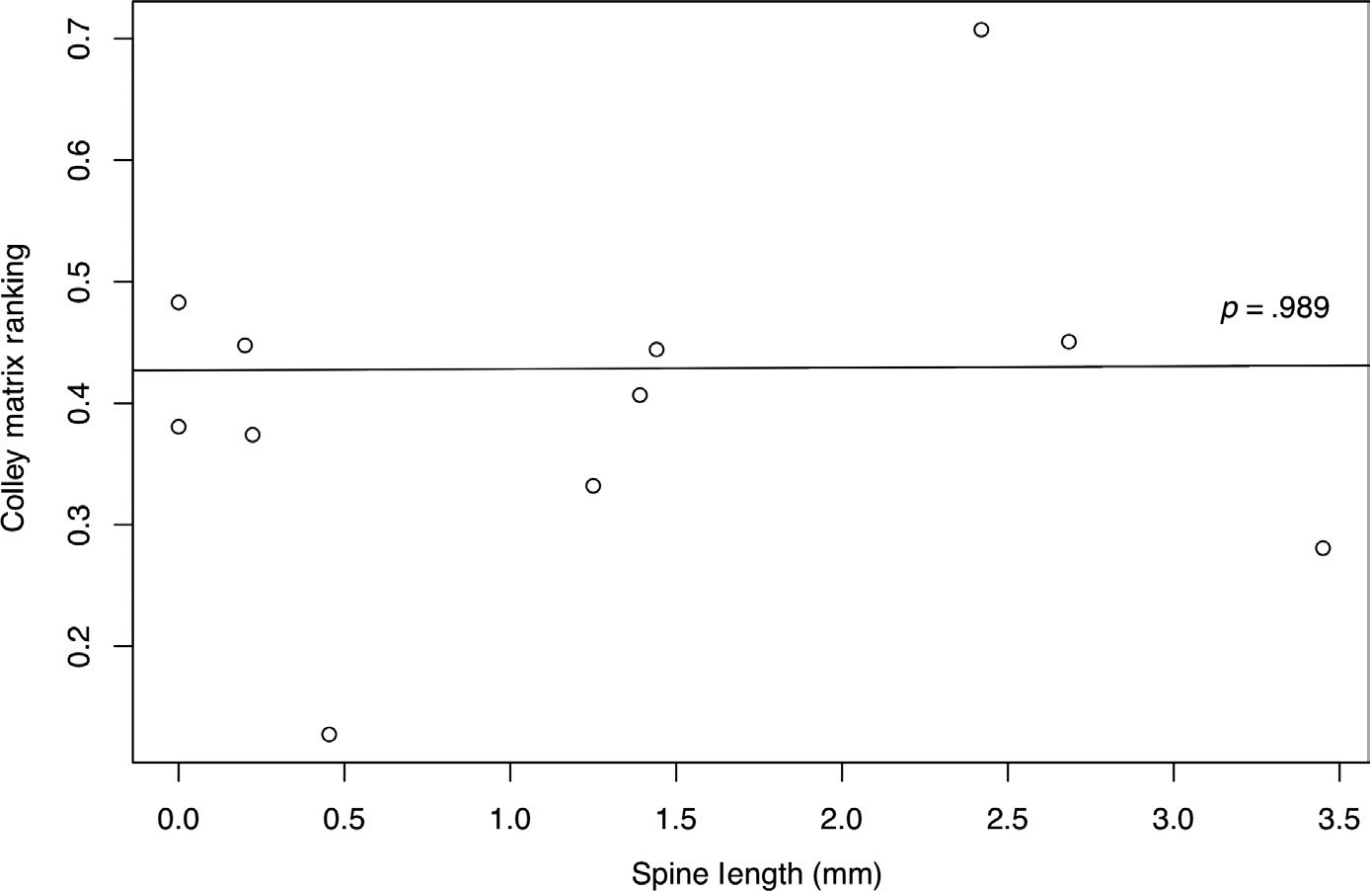
Competitive ability results from multispecies trials (*n* = 11) using a phylogenetic regression along Colley Matrix scores

**FIGURE 5:**
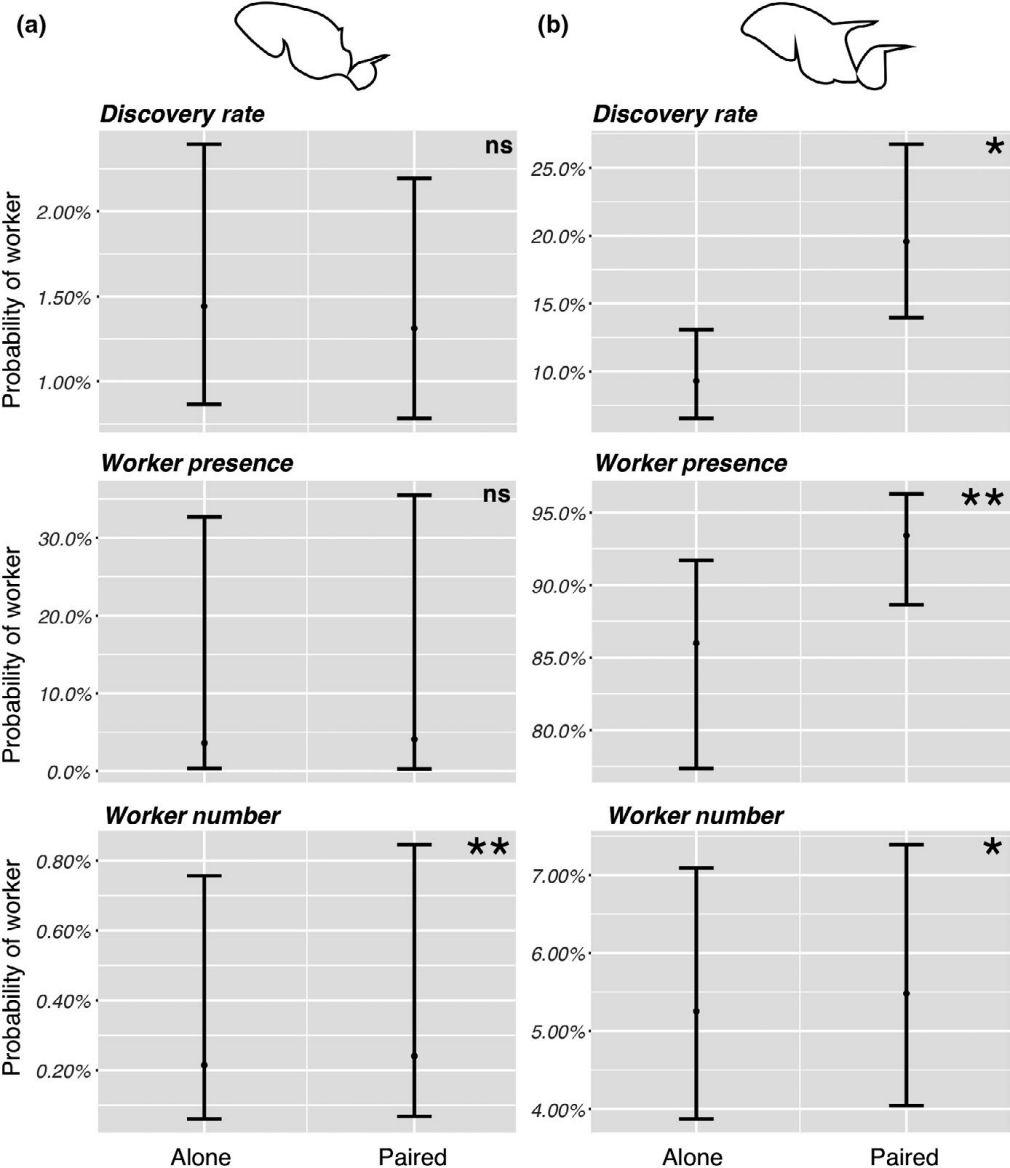
Resource discovery rate and foraging effort (“Alone”) outcomes compared to competitive ability outcomes (“Paired”) for (a) *P. flavicornis* and (b) *P. laevigata*. Bar lines indicate boundaries of the 95% prediction interval, that is, the interval expected to contain 95% of future observations for a given species. *p < .01, **p < .001, ns,not significant (*p* > .1)

Our phylogenetic regression of Colley Matrix values across spine length for the multispecies trials did not support any significant association (*p* = .89, Figure 4, Table S5). In our two‐species trials (Table S6), our results differed for each species. For *P. flavicornis*, the species with smaller spines, we found no significant difference between alone and paired trials for Discovery Rate (*Z* = 0.27, *p* = .79) or Worker Presence (*Z* = −1.34, *p* = .18) but did find higher Worker Number in the paired trials (*Z* = −3.84, *p* < .01) (Figure 5a). For *P. laevigata*, the species with longer spines, we found significant differences across all trial comparisons, with faster Discovery Rate (*Z* = −3.05, *p* < .01) and higher Worker Presence (*Z* = −7.58, *p* < .01) and Worker Number (*Z* = −2.97, *p* < .01) in paired versus alone trials (Figure 5b).

### 3.3 Antipredator defense

**FIGURE 6:**
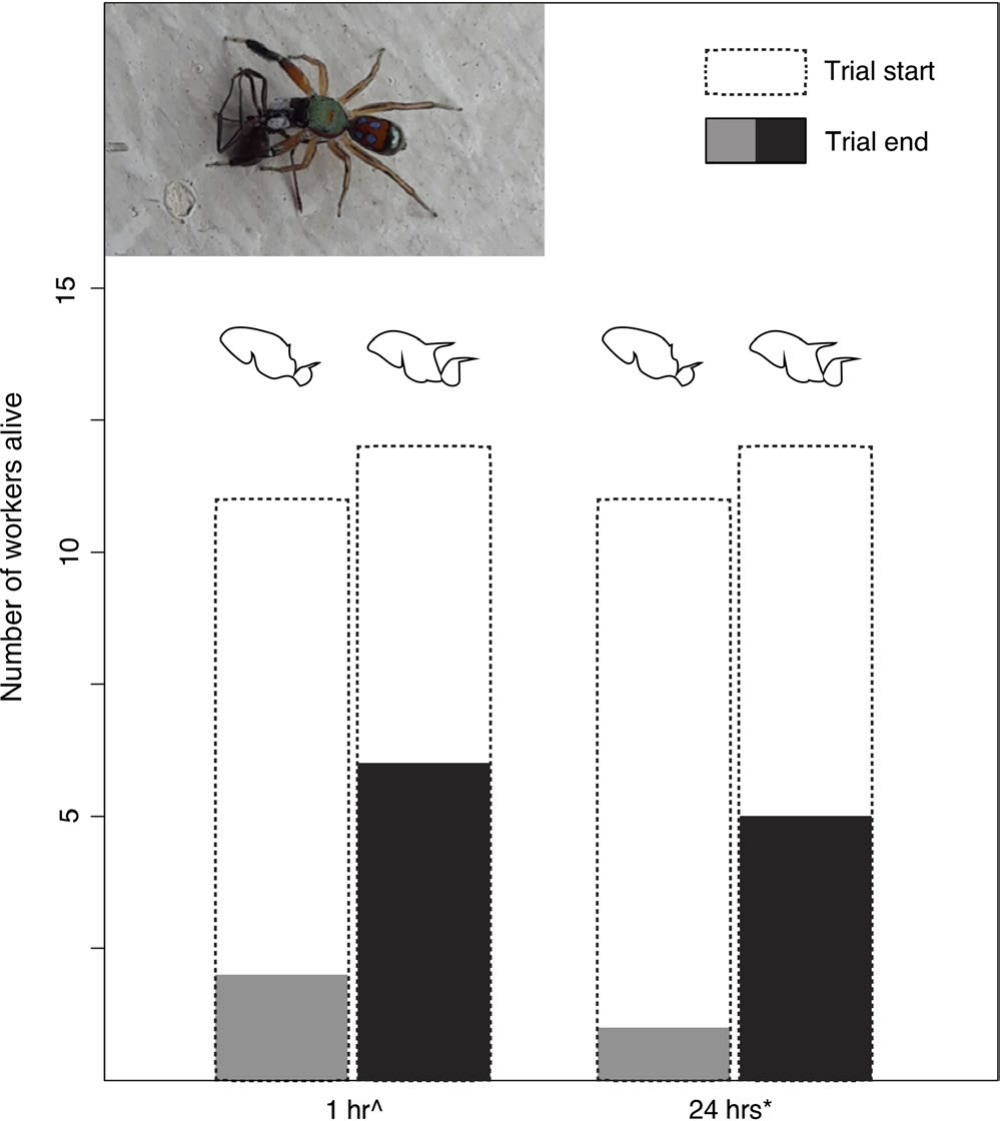
Differences in predator–prey survival for *P. flavicornis* and *P. laevigata* workers presented to the jumping spider *S. semiglaucus*. Chi‐square: ^*p*= .110, *p = .076

We found that survival from predation by the spider *S. semiglaucus* tended to be higher for *P. laevigata*, the species with longer spines, than for *P. flavicornis*. This relationship was not statistically significant after one hour (*χ* ^2^ = 2.56, *p* = .11), but was marginally significant after 24 hr (*χ* ^2^ = 3.16, *p* = .08) (Figure 6).

## 4 DISCUSSION

We tested potential ecological benefits and costs of cuticular spines, a widespread and variable defensive morphological trait in ants. We found general support for a neutral to positive relationship between spines and all ecological traits we tested, including resource discovery rate, foraging effort, competitive ability, and antipredator defense, with no costs detected. Our results thus support spines as a key defensive trait promoting broad ecological success and do not support expectations of the ecological trade‐off hypothesis. To our knowledge, this positive association between spines and resource acquisition traits and (potentially) competitive ability, not just antipredator defense, has not been previously proposed in the literature and represents an intriguing area of future research.

Our finding of broader ecological trait benefits of an adaptive trait beyond the most proximate adaptive function highlights the wide‐ranging potential impacts of morphological trait evolution. We should note that, in fact, the support for a positive association between spines and defense against our invertebrate jumping spider predator was rather modest (Figure 6), which is consistent with the expectation stated by Pekár et al. (2017) that spines should not be very effective against invertebrate as opposed to vertebrate predators (e.g., Ito et al., 2016). On the other hand, the strongest result was a positive association between spines and foraging ability (Figure 3). This dynamic is similar to other systems where release or escape from one selection pressure allows a species to invest in traits adapted to other selection pressures (Fulton, Wainwright, Hoey, & Bellwood, 2016; Kinnison, Unwin, & Quinn, 2003; Kitajima, 1994). Our study suggests a potential mechanistic sequence that results in spines driving broad ecological success: Spines first confer antipredator defense (against both vertebrate and invertebrate predators, or primarily vertebrate predators), which then reduces the need to hide from predators, subsequently allowing for more conspicuous foraging, which facilitates faster resource discovery and enhanced resource dominance, further conferring competitive advantages relative to less spinescent populations.

While we do not test trait‐based diversification here, recent phylogenetic work on spines in ants suggests spines are an important morphological trait in ant evolution. One study proposed a link between extreme spinescence and occupancy of inland niches on islands in the hyperdiverse genus *Pheidole* (Sarnat & Moreau, 2011), while later work supported a link between spines and islands but not high‐elevation habitats (Sarnat et al., 2017). Blanchard and Moreau (2017) demonstrated a positive association between spines and elevated diversification rates as well as high evolutionary lability for spines across the ant phylogeny. Considered together with these studies and previous work supporting ant spines as protection against vertebrate predators (Ito et al., 2016), our results suggest that spinescence serves as a generalized physical defensive trait with broad downstream ecological benefits potentially promoting high diversification rates in *Polyrhachis*, the fourth most diverse ant genus with 700 described species, and ants more broadly. However, more trait‐based phylogenetic work is needed to detail the importance of spines for ant diversification, and in particular more robustly establish possible ecological mechanisms, like escape‐and‐radiate dynamics (Arbuckle & Speed, 2015; Ehrlich & Raven, 1964), linking defensive function and evolutionary radiation.

Although ecological trade‐offs exist in many taxa and especially between competitive ability and antipredator defense (Jessup & Bohannan, 2008; Kneitel & Chase, 2004), we did not find any spine‐mediated trade‐off between antipredator defense and resource discovery rate, foraging effort, or competitive ability. The production of cuticular spines may be energetically cheap and/or developmentally simple, which may also explain the high evolutionary lability of the trait (Blanchard & Moreau, 2017), and future work could focus on a more mechanistic understanding of spine development and potential costs. In contrast to some plant species which exhibit phenotypic plasticity in increasing spine production when induced by herbivores (e.g., Young, 1987), spine production is canalized among the adult worker caste at a colonial level, possibly owing to consistency of predator pressure or low costs of trait production. Additionally, although competition and predation are fundamental ecological processes, other dynamics may exert greater constraining influences that would explain why, given their apparent broad benefits, spines have not evolved in all ant species and are, for example, largely unique to *Polyrhachis* among the Formicinae subfamily. Murrell and Juliano (2013) found that a competition–predation trade‐off is not supported in some mosquitoes, instead finding a colonization–competition trade‐off. Supriya, Price, and Rowe (2018) report a positive, rather than negative, correlation between pre‐ and postcopulatory traits in warblers, possibly resulting from differential investment in overall fertilization success versus survival. Thus, a trade‐off may exist between competition or predation and traits not included in our study, such as number of reproductives or colony longevity. Still, given the prevalence of a competition–predation trade‐off across many terrestrial and aquatic systems (Kneitel & Chase, 2004), our failure to detect evidence of such a trade‐off is intriguing and warrants further study.

It is important to recognize that our experimental methods, in an effort to render the system tractable, also simplify the pressures that exist in a highly variable natural environment. Adding the structural complexity of the physical environment, including vertebrate predators, and allowing workers to compete in a less‐restricted spatial context may reveal dynamics that our methods could not detect. Furthermore, in “controlling for” colony size, we may also artificially constrain a species’ competitive ability that would normally compensate for smaller spines by having a larger colony size. Nevertheless, we view this study as a first step toward identifying mechanistic explanations for spines and ecological success in this group, and a good starting point for developing more complex theories and experimental methods.

Future research should target predator‐driven dynamics potentially influencing morphological divergence as well as other ecological and evolutionary processes in ants. Such top‐down influences on ant communities and species have been underexplored (Cerdá et al., 2013), and our study supports the importance of predator–prey dynamics for ants. Although difficult to execute, more sophisticated functional trait studies, like those seen in plants, will be necessary to test theories positing trait‐based explanatory factors for community assembly as well as ecological and evolutionary success in ants and other diverse animal groups. Multispecies and multitrait approaches, and in particular controlled trials with experimental manipulation of traits (Larabee & Suarez, 2015), are sure to enhance our understanding of the link between defensive traits, species interactions, and diversification in these groups. Most broadly, exploring the wider ecological implications of functional traits beyond their proximate adaptive function will promote a deeper understanding of the impacts of morphological traits in animal ecology.

## CONFLICT OF INTEREST

The authors have no competing interests.

## AUTHOR CONTRIBUTION

**Benjamin David Blanchard:** Conceptualization (lead); Data curation (lead); Formal analysis (lead); Funding acquisition (lead); Investigation (lead); Methodology (lead); Project administration (lead); Visualization (lead); Writing‐original draft (lead). **Akihiro Nakamura:** Conceptualization (supporting); Funding acquisition (supporting); Project administration (supporting); Resources (supporting); Supervision (equal); Writing‐review & editing (supporting). **Min Cao:** Conceptualization (supporting); Funding acquisition (supporting); Resources (lead); Supervision (supporting); Writing‐review & editing (supporting). **Stephanie T Chen:** Methodology (supporting); Writing‐review & editing (supporting). **Corrie S. Moreau:** Conceptualization (supporting); Funding acquisition (supporting); Methodology (supporting); Project administration (supporting); Resources (supporting); Supervision (equal); Writing‐review & editing (supporting) .

## Supporting information

Supplementary Material

## Data Availability

All data files are archived in the Dryad data repository at https://doi.org/10.5061/dryad.d51c5b004 as well as https://github.com/BenjaminBlanchard/spineanddine.
