# Supplementary material for: Spine and dine: A key defensive trait promotes ecological success in spiny ants
Source: Ecol Evol. 2020 Apr 29;10(12):5852–63. doi: 10.1002/ece3.6322 (PMC7319116; doi:10.1002/ece3.6322)
Supplement: Supplementary file 1 — Supplementary Material [file ECE3-10-5852-s001.pdf]

SUPPORTING INFORMATION FIGURES AND TABLES

Figure S1: Experimental design for resource discovery rate and foraging effort trials. Image credit: B.D. Blanchard.

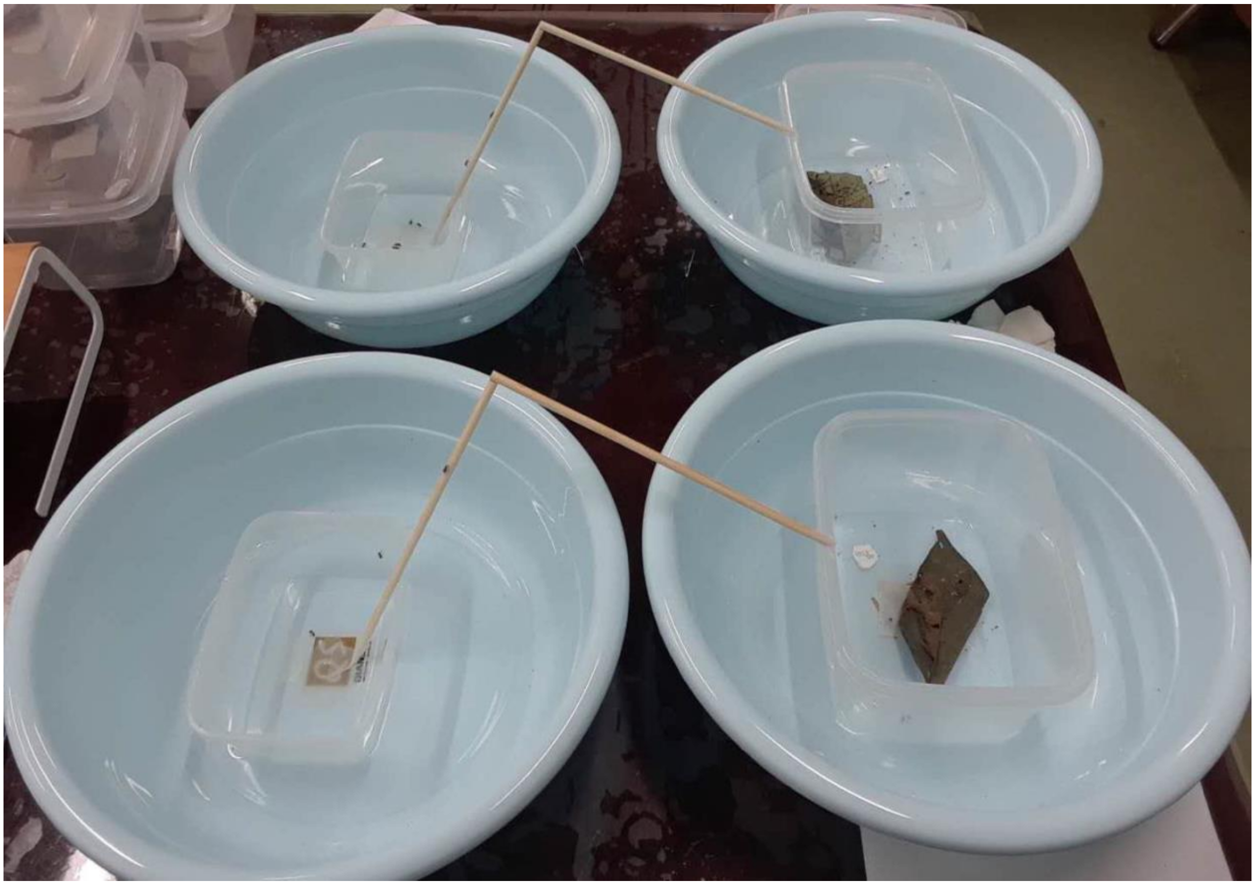

Figure S2: Resource discovery rate and foraging effort results for the multi-species (n = 11) trials with *P. (Cyrtomyrma)* species (n = 2) included. ns = not significant (p > 0.1). Grey zones indicate the boundaries of the 95% prediction interval, i.e. the interval expected to contain 95% of future observations for a given spine length.

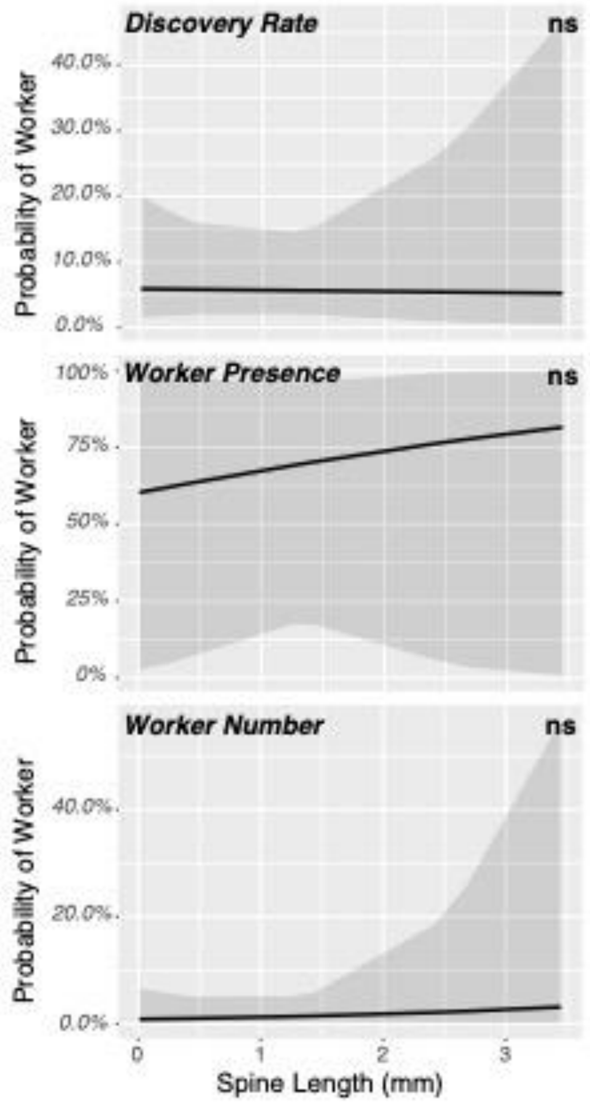

Table S1: Colony information for multi-species trials. For morphometric measurements, one representative worker was used, as *Polyrhachis* species are monomorphic with intraspecific variance much smaller than interspecific variance. ns = not significant ($p > 0.1$).

Colony Acc. #	Species	Subgenus	Collection Date	Competition pair	Colony Size (# Workers), Resource Discovery Time/Foraging Effort	Colony Size (# Workers), Competitive Ability	Body Size (mm)
BDB 703	P_rastellata	Cyrtomyrma	Jun/28/2017	BDB 707	79	79	1.88
BDB 707	P_bicolor	Myrmhopla	Jun/28/2017	BDB 703	41	41	1.946
BDB 708	P_armata	Myrmhopla	Jun/28/2017	BDB 709	45	45	4
BDB 709	P_laevigata	Myrmhopla	Jun/28/2017	BDB 708	16	16	1.843
BDB 710	P_furcata	Myrmhopla	Jun/28/2017	BDB 725	315	315	1.851
BDB 711	P_laevigata	Myrmhopla	Jun/28/2017	BDB 712	82	82	1.843
BDB 712	P_flavicornis	Myrmatopa	Jun/28/2017	BDB 711	65	61	1.744
BDB 713	P_flavicornis	Myrmatopa	Jun/28/2017	BDB 726	25	25	1.744
BDB 714	P_flavicornis	Myrmatopa	Jun/28/2017	BDB 719	5	5	1.744
BDB 719	P_rastellata	Cyrtomyrma	Jun/29/2017	BDB 714	26	26	1.88
BDB 720	P_emmae	Myrmhopla	Jun/29/2017	BDB 724	86	86	1.7
BDB 724	P_armata	Myrmhopla	Jun/29/2017	BDB 720	32	32	4
BDB 725	P_armata	Myrmhopla	Jun/29/2017	BDB 710	51	51	4
BDB 726	P_rastellata	Cyrtomyrma	Jun/29/2017	BDB 713	21	21	1.88
BDB 727	P_armata	Myrmhopla	Jun/29/2017	BDB 732	92	92	4
BDB 728	P_armata	Myrmhopla	Jun/29/2017	BDB 730	22	22	4
BDB 729	P_flavicornis	Myrmatopa	Jun/29/2017	BDB 731	130	130	1.744
BDB 730	P_flavicornis	Myrmatopa	Jun/30/2017	BDB 728	69	69	1.744
BDB 731	P_furcata	Myrmhopla	Jun/30/2017	BDB 729	105	90	1.851
BDB 732	P_furcata	Myrmhopla	Jun/30/2017	BDB 727	141	119	1.851
BDB 733	P_furcata	Myrmhopla	Jun/30/2017	BDB 735	164	154	1.851
BDB 734	P_flavicornis	Myrmatopa	Jun/30/2017	N/A	2	N/A	1.744
BDB 735	P_flavicornis	Myrmatopa	Jun/30/2017	BDB 733	71	71	1.744
BDB 736	P_flavicornis	Myrmatopa	Jun/30/2017	N/A	18	N/A	1.744
BDB 737	P_laevigata	Myrmhopla	Jun/30/2017	BDB 739	38	38	1.843
BDB 738	P_bicolor	Myrmhopla	Jun/30/2017	BDB 741	37	37	1.946
BDB 739	P_flavicornis	Myrmatopa	Jun/30/2017	BDB 737	47	47	1.744
BDB 740	P_bicolor	Myrmhopla	Jun/30/2017	BDB 744	111	111	1.946
BDB 741	P_furcata	Myrmhopla	Jun/30/2017	BDB 738	473	473	1.851
BDB 742	P_flavicornis	Myrmatopa	Jun/30/2017	BDB 748	15	15	1.744
BDB 743	P_flavicornis	Myrmatopa	Jun/30/2017	BDB 746	17	17	1.744
BDB 744	P_flavicornis	Myrmatopa	Jun/30/2017	BDB 740	24	24	1.744

BDB 745	P_rotoccipita	Cyrtomyrma	Jun/30/2017	BDB 747	61	61	1.91
BDB 746	P_leviuscula	Myrmatopa	Jun/30/2017	BDB 743	6	6	2.1
BDB 747	P_flavicornis	Myrmatopa	Jun/30/2017	BDB 745	92	92	1.744
BDB 748	P_rotoccipita	Cyrtomyrma	Jun/30/2017	BDB 742	71	71	1.91
BDB 751	P_rotoccipita	Cyrtomyrma	Jun/30/2017	BDB 763	53	53	1.91
BDB 753	P_bicolor	Myrmhopla	Jul/01/2017	BDB 762	61	61	1.946
BDB 754	P_furcata	Myrmhopla	Jul/01/2017	BDB 760	820	720	1.851
BDB 755	P_flavicornis	Myrmatopa	Jul/01/2017	N/A	37	N/A	1.744
BDB 756	P_flavicornis	Myrmatopa	Jul/01/2017	N/A	20	N/A	1.744
BDB 757	P_flavicornis	Myrmatopa	Jul/01/2017	N/A	3	N/A	1.744
BDB 758	P_flavicornis	Myrmatopa	Jul/01/2017	N/A	22	N/A	1.744
BDB 759	P_flavicornis	Myrmatopa	Jul/03/2017	N/A	65	N/A	1.744
BDB 760	P_flavicornis	Myrmatopa	Jul/03/2017	BDB 754	36	16	1.744
BDB 761	P_flavicornis	Myrmatopa	Jul/03/2017	N/A	36	N/A	1.744
BDB 762	P_rotoccipita	Cyrtomyrma	Jul/03/2017	BDB 753	44	43	1.91
BDB 763	P_laevigata	Myrmhopla	Jul/03/2017	BDB 751	30	30	1.843
BDB 764	P_laevigata	Myrmhopla	Jul/03/2017	BDB 780	63	63	1.843
BDB 765	P_armata	Myrmhopla	Jul/03/2017	BDB 769	55	55	4
BDB 766	P_flavicornis	Myrmatopa	Jul/03/2017	BDB 767	55	55	1.744
BDB 767	P_armata	Myrmhopla	Jul/03/2017	BDB 766	55	55	4
BDB 768	P_laevigata	Myrmhopla	Jul/03/2017	BDB 772	218	218	1.843
BDB 769	P_flavicornis	Myrmatopa	Jul/03/2017	BDB 765	189	189	1.744
BDB 770	P_flavicornis	Myrmatopa	Jul/03/2017	BDB 771	35	35	1.744
BDB 771	P_laevigata	Myrmhopla	Jul/03/2017	BDB 770	22	22	1.843
BDB 772	P_furcata	Myrmhopla	Jul/03/2017	BDB 768	103	103	1.851
BDB 778	P_flavicornis	Myrmatopa	July/05/2017	N/A	23	N/A	1.744
BDB 779	P_flavicornis	Myrmatopa	July/05/2017	N/A	11	N/A	1.744
BDB 780	P_armata	Myrmhopla	July/10/2017	BDB 764	53	53	4
BDB 781	P_flavicornis	Myrmatopa	July/10/2017	BDB 783	59	59	1.744
BDB 783	P_furcata	Myrmhopla	July/10/2017	BDB 781	247	247	1.851
BDB 784	P_flavicornis	Myrmatopa	July/10/2017	N/A	28	N/A	1.744
BDB 785	P_leviuscula	Myrmatopa	July/10/2017	N/A	15	N/A	2.1
BDB 786	P_leviuscula	Myrmatopa	July/10/2017	N/A	17	N/A	2.1
BDB 787	P_leviuscula	Myrmatopa	July/10/2017	N/A	30	N/A	2.1
BDB 788	P_rotoccipita	Cyrtomyrma	July/10/2017	BDB 792	44	44	1.91
BDB 789	P_flavicornis	Myrmatopa	July/10/2017	N/A	54	N/A	1.744
BDB 790	P_flavicornis	Myrmatopa	July/10/2017	BDB 793	32	32	1.744
BDB 791	P_flavicornis	Myrmatopa	July/10/2017	N/A	92	N/A	1.744
BDB 792	P_laevigata	Myrmhopla	July/10/2017	BDB 788	102	102	1.843
BDB 793	P_laevigata	Myrmhopla	July/10/2017	BDB 790	45	45	1.843

BDB 794	P_rufipes	Myrmhopla	July/10/2017	BDB 803	117	117	2.05
BDB 795	P_furcata	Myrmhopla	July/10/2017	BDB 801	224	224	1.851
BDB 796	P_laevigata	Myrmhopla	July/10/2017	BDB 807	47	47	1.843
BDB 797	P_furcata	Myrmhopla	July/10/2017	BDB 802	316	301	1.851
BDB 798	P_furcata	Myrmhopla	July/10/2017	BDB 804	37	37	1.851
BDB 800	P_flavicornis	Myrmatopa	July/12/2017	BDB 806	3	3	1.744
BDB 801	P_laevigata	Myrmhopla	July/12/2017	BDB 795	82	82	1.843
BDB 802	P_laevigata	Myrmhopla	July/12/2017	BDB 797	72	72	1.843
BDB 803	P_laevigata	Myrmhopla	July/12/2017	BDB 794	155	155	1.843
BDB 804	P_laevigata	Myrmhopla	July/12/2017	BDB 798	29	29	1.843
BDB 806	P_bicolor	Myrmhopla	July/25/2017	BDB 800	9	9	1.946
BDB 807	P_bicolor	Myrmhopla	July/25/2017	BDB 796	34	34	1.946
BDB 808	P_bicolor	Myrmhopla	July/25/2017	BDB 809	39	39	1.946
BDB 809	P_armata	Myrmhopla	July/25/2017	BDB 808	43	43	4
BDB 810	P_thrinax	Myrmothrinax	July/25/2017	BDB 811	46	46	2.006
BDB 811	P_laevigata	Myrmhopla	July/25/2017	BDB 810	125	125	1.843
BDB 813	P_laevigata	Myrmhopla	July/29/2017	N/A	112	N/A	1.843

Table S2: Colony information for two-species trials (*P. flavicornis* and *P. laevigata*).

Colony Acc. #	Species	Collection Date	Competition pair	Colony Size (# Workers), Resource Discovery Rate/Foraging Effort	Colony Size (# Workers), Competitive Ability
BDB 814	P. flavicornis	Jun/27/2018	BDB 825	54	53
BDB 825	P. laevigata	Jun/28/2018	BDB 814	126	126
BDB 815	P. flavicornis	Jun/27/2018	BDB 847	23	21
BDB 847	P. laevigata	Jun/29/2018	BDB 815	145	145
BDB 816	P. flavicornis	Jun/27/2018	BDB 826	48	47
BDB 826	P. laevigata	Jun/28/2018	BDB 816	24	24
BDB 817	P. flavicornis	Jun/27/2018	BDB 821	19	19
BDB 821	P. laevigata	Jun/28/2018	BDB 817	83	83
BDB 818	P. flavicornis	Jun/27/2018	BDB 819	34	34
BDB 819	P. laevigata	Jun/28/2018	BDB 818	37	37
BDB 820	P. flavicornis	Jun/28/2018	BDB 838	37	37
BDB 838	P. laevigata	Jun/29/2018	BDB 820	41	39
BDB 827	P. flavicornis	Jun/28/2018	BDB 840	43	42
BDB 840	P. laevigata	Jun/29/2018	BDB 827	30	30
BDB 828	P. flavicornis	Jun/28/2018	BDB 852	62	62
BDB 852	P. laevigata	Jun/30/2018	BDB 828	21	21
BDB 830	P. flavicornis	Jun/29/2018	BDB 868	121	121
BDB 868	P. laevigata	Jun/30/2018	BDB 830	26	26
BDB 831	P. flavicornis	Jun/29/2018	BDB 851	66	64
BDB 851	P. laevigata	Jun/30/2018	BDB 831	75	74
BDB 832	P. flavicornis	Jun/29/2018	BDB 869	35	35
BDB 869	P. laevigata	Jun/30/2018	BDB 832	42	40
BDB 833	P. flavicornis	Jun/29/2018	BDB 850	67	67
BDB 850	P. laevigata	Jun/30/2018	BDB 833	46	46
BDB 834	P. flavicornis	Jun/29/2018	BDB 866	167	151
BDB 866	P. laevigata	Jun/30/2018	BDB 834	141	132
BDB 835	P. flavicornis	Jun/29/2018	BDB 846	59	58
BDB 846	P. laevigata	Jun/29/2018	BDB 835	74	73
BDB 836	P. flavicornis	Jun/29/2018	BDB 856	12	12
BDB 856	P. laevigata	Jun/30/2018	BDB 836	196	196
BDB 837	P. flavicornis	Jun/29/2018	BDB 871	11	11
BDB 871	P. laevigata	Jun/30/2018	BDB 837	7	7
BDB 839	P. flavicornis	Jun/29/2018	BDB 863	181	161

BDB 863	P. laevigata	Jun/30/2018	BDB 839	131	130
BDB 841	P. flavicornis	Jun/29/2018	BDB 857	34	34
BDB 857	P. laevigata	Jun/30/2018	BDB 841	82	82
BDB 842	P. flavicornis	Jun/29/2018	BDB 855	42	41
BDB 855	P. laevigata	Jun/30/2018	BDB 842	48	48
BDB 843	P. flavicornis	Jun/29/2018	BDB 858	46	46
BDB 858	P. laevigata	Jun/30/2018	BDB 843	141	141
BDB 845	P. flavicornis	Jun/29/2018	BDB 867	44	43
BDB 867	P. laevigata	Jun/30/2018	BDB 845	144	143
BDB 848	P. flavicornis	Jun/29/2018	BDB 860	63	63
BDB 860	P. laevigata	Jun/30/2018	BDB 848	96	96
BDB 849	P. flavicornis	Jun/29/2018	BDB 859	36	36
BDB 859	P. laevigata	Jun/30/2018	BDB 849	65	65
BDB 853	P. flavicornis	Jun/30/2018	BDB 844	25	25
BDB 844	P. laevigata	Jun/29/2018	BDB 853	79	78
BDB 873	P. flavicornis	Jul/02/2018	BDB 870	108	110
BDB 870	P. laevigata	Jun/30/2018	BDB 873	110	108
BDB 874	P. flavicornis	Jul/02/2018	BDB 865	54	38
BDB 865	P. laevigata	Jun/30/2018	BDB 874	118	117
BDB 875	P. flavicornis	Jul/02/2018	BDB 861	33	33
BDB 861	P. laevigata	Jun/30/2018	BDB 875	116	116
BDB 876	P. flavicornis	Jul/02/2018	BDB 862	42	41
BDB 862	P. laevigata	Jun/30/2018	BDB 876	199	198
BDB 877	P. flavicornis	Jul/02/2018	BDB 864	37	34
BDB 864	P. laevigata	Jun/30/2018	BDB 877	108	107
BDB 878	P. flavicornis	Aug/9/2018	BDB 881	51	51
BDB 881	P. laevigata	Aug/9/2018	BDB 878	40	39

Table S3: Correlation tables produced using the R function “cor” (Pearson correlation coefficient). All variables included in GLMs/GLMMs exhibit values < 0.8. Note that “Discovered” is not a variable but instead used to set the Geometric model. See https://github.com/BenjaminBlanchard/spineanddine for R scripts.

Two-Species - Resource Discovery Rate

	Time_Disc	Discovered	Col_Size
Time_Disc	1.000	-0.905	-0.253
Discovered	-0.905	1.000	0.345
Col_Size	-0.253	0.345	1.000

Two-Species - Foraging Effort (Worker Presence/Worker Number)

	Time_Bin	Worker Number	Worker Worker Presence	Col_Size
Time_Bin	1.000	0.187	0.181	0.000
Worker Number	0.187	1.000	0.522	0.497
Worker Worker Presence	0.181	0.522	1.000	0.276
Col_Size	0.000	0.497	0.276	1.000

Two-Species - Competition (P. flavicornis; Discovery Rate)

	Time_Disc	Discovered	Col_Size
Time_Disc	1.000	-0.944	-0.421
Discovered	-0.944	1.000	0.402
Col_Size	-0.421	0.402	1.000

Two-Species - Competition (P. laevigata; Discovery Rate)

	Time_Disc	Discovered	Col_Size
Time_Disc	1.000	-0.511	0.102
Discovered	-0.511	1.000	0.110
Col_Size	0.102	0.110	1.000

Two-Species - Competition (P. flavicornis; Worker Presence/Worker Number)

	Time_Bin	Worker Number	Worker Worker Presence	Col_Size
Time_Bin	1.000	0.217	0.214	0.000
Worker Number	0.217	1.000	0.635	0.487
Worker Worker Presence	0.214	0.635	1.000	0.339
Col_Size	0.000	0.487	0.339	1.000

Two-Species - Competition (P. laevigata; Worker Presence/Worker Number)

	Time_Bin	Worker Number	Worker Worker Presence	Col_Size
Time_Bin	1.000	0.102	0.085	0.000
Worker Number	0.102	1.000	0.408	0.461
Worker Worker Presence	0.085	0.408	1.000	0.086
Col_Size	0.000	0.461	0.086	1.000

Multi-Species - Resource Discovery Rate

	Body	Spine	Time_Disc	Discovered	Col_Size
Body	1.000	0.685	-0.331	0.298	-0.086
Spine	0.685	1.000	-0.495	0.470	0.362
Time_Disc	-0.331	-0.495	1.000	-0.887	-0.262
Discovered	0.298	0.470	-0.887	1.000	0.217
Col_Size	-0.086	0.362	-0.262	0.217	1.000

Multi-Species - Resource Discovery Rate (No *P. (Cyrtomyrma)* spp.)

	Body	Spine	Time_Disc	Discovered	Col_Size
Body	1.000	0.708	-0.368	0.321	-0.093
Spine	0.708	1.000	-0.667	0.597	0.357
Time_Disc	-0.368	-0.667	1.000	-0.883	-0.300
Discovered	0.321	0.597	-0.883	1.000	0.241
Col_Size	-0.093	0.357	-0.300	0.241	1.000

Multi-Species - Foraging Effort (Worker Presence/Worker Number)

	Body	Spine	Time_Bin	Worker Number	Worker Worker Presence	Col_Size
Body	1.000	0.685	0.000	0.001	0.259	-0.086
Spine	0.685	1.000	0.000	0.258	0.386	0.362
Time_Bin	0.000	0.000	1.000	0.058	0.066	0.000
Worker Number	0.001	0.258	0.058	1.000	0.338	0.739
Worker Worker Presence	0.259	0.386	0.066	0.338	1.000	0.241
Col_Size	-0.086	0.362	0.000	0.739	0.241	1.000

Multi-Species - Foraging Effort (Worker Presence/Worker Number; No *P. (Cyrtomyrma)* spp.)

	Body	Spine	Time_Bin	Worker Number	Worker Worker Presence	Col_Size
Body	1.000	0.708	0.000	0.004	0.299	-0.093
Spine	0.708	1.000	0.000	0.295	0.563	0.357
Time_Bin	0.000	0.000	1.000	0.059	0.070	0.000
Worker Number	0.004	0.295	0.059	1.000	0.341	0.749

Worker Worker Presence	0.299	0.563	0.070	0.341	1.000	0.284
Col_Size	-0.093	0.357	0.000	0.749	0.284	1.000

Table S4: GLMM results for multi-species and two-species resource discovery rate and foraging effort trials. For the two-species results, the “Species” variable estimates are displayed for *P. laevigata* trials with *P. flavicornis* trial values treated as the baseline.

Multi-Species GLM/GLMM results (n = 9)

**Trial**	**Variable**	**Estimate**	**Std. Error**	**z-score**	***p*-value**
Multi-Species [n = 9]					
Discovery Rate	Spine Length	0.759	0.502	1.511	0.131
	Body Size	0.045	0.757	0.060	0.952
	Colony Size	3.692	1.453	2.541	**0.011**
Worker Presence	Spine Length	2.591	1.197	2.165	**0.030**
	Body Size	-0.664	1.739	-0.382	0.7026
	Time Bin	1.459	0.178	8.186	**<0.001**
	Colony Size	6.245	3.961	1.577	0.115
Worker Number	Spine Length	1.425	0.821	1.735	0.083
	Body Size	-0.318	1.233	-0.258	0.797
	Time Bin	0.755	0.031	24.592	**<0.001**
Two-Species					
Discovery Rate	Species	1.691	0.364	4.645	**<0.001**
	Colony Size	0.001	0.003	0.202	0.84
Worker Presence	Species	3.659	1.050	3.485	**0.001**
	Time Bin	3.780	0.240	15.738	**<0.001**
	Colony Size	0.026	0.011	2.400	**0.016**
Worker Number	Species	2.705	0.611	4.425	**<0.001**
	Time Bin	1.127	0.034	32.942	**<0.001**
Multi-Species [n = 11, including *P. (Cyrtomyrma)* species]					
Discovery Rate	Spine Length	-0.036	0.527	-0.069	0.945
	Body Size	0.597	0.933	-0.640	0.522
	Colony Size	3.981	1.472	2.705	**0.007**
Worker Presence	Spine Length	0.311	1.355	0.230	0.818
	Body Size	0.925	2.377	0.389	0.698
	Time Bin	1.473	0.175	8.395	**<0.001**
	Colony Size	7.069	4.047	1.747	0.081
Worker Number	Spine Length	0.389	0.753	0.516	0.606
	Body Size	0.370	1.336	0.277	0.782
	Time Bin	0.677	0.029	23.652	**<0.001**

Table S5: Data and matrices used to construct Colley Matrix, and analysis results.

	P_orbihumera	P_bicolor	P_orbihumera (Sum)	P_bicolor (Sum)
Average in central chamber	0.03734	0.03902	0.06034	0.07236
Average in opponent's chamber	0.02300	0.03333		
Colony size	79.00000	41.00000		

	P_armata	P_laevigata	P_armata (Sum)	P_laevigata (Sum)
Average in central	0.03593	0.05208	0.07037	0.05208
Average in opponent	0.03444	0.00000		
Colony size	45.00000	16.00000		

	P_furcata	P_armata	P_furcata (Sum)	P_armata (Sum)
Average in central	0.23074	0.01863	0.30106	0.02190
Average in opponent	0.07032	0.00327		
Colony size	315.00000	51.00000		

	P_laevigata	P_flavicornis	P_laevigata (Sum)	P_flavicornis (Sum)
Average in central	0.04492	0.00519	0.04492	0.00574
Average in opponent	0.00000	0.00055		
Colony size	82.00000	61.00000		

	P_flavicornis	P_orbihumera	P_flavicornis (Sum)	P_orbihumera (Sum)
Average in central	0.00000	0.11090	0.00000	0.14359
Average in opponent	0.00000	0.03269		
Colony size	5.00000	26.00000		

	P_emmae	P_armata	P_emmae (Sum)	P_armata (Sum)
Average in central	0.07035	0.21198	0.13430	0.30365
Average in opponent	0.06395	0.09167		
Colony size	86.00000	32.00000		

	P_flavicornis	P_orbihumera	P_flavicornis (Sum)	P_orbihumera (Sum)
Average in central	0.00000	0.11190	0.00000	0.12937
Average in opponent	0.00000	0.01746		
Colony size	25.00000	21.00000		

	P_armata	P_flavicornis	P_armata (Sum)	P_flavicornis (Sum)
Average in central	0.09091	0.03692	0.15076	0.04628
Average in opponent	0.05985	0.00936		
Colony size	22.00000	130.00000		

	P_flavicornis	P_furcata	P_flavicornis (Sum)	P_furcata (Sum)
Average in central	0.05615	0.01759	0.06910	0.01870
Average in opponent	0.01295	0.00111		
Colony size	130.00000	90.00000		

	P_armata	P_furcata	P_armata (Sum)	P_furcata (Sum)
Average in central	0.01993	0.08389	0.16431	0.09342
Average in opponent	0.14438	0.00952		
Colony size	92.00000	119.00000		

	P_furcata	P_flavicornis	P_furcata (Sum)	P_flavicornis (Sum)
Average in central	0.14275	0.00845	0.19838	0.00845
Average in opponent	0.05563	0.00000		
Colony size	154.00000	71.00000		

	P_laevigata	P_flavicornis	P_laevigata (Sum)	P_flavicornis (Sum)
Average in central	0.00000	0.00000	0.00000	0.00000
Average in opponent	0.00000	0.00000		
Colony size	38.00000	47.00000		

	P_bicolor	P_furcata	P_bicolor (Sum)	P_furcata (Sum)
Average in central	0.08153	0.03393	0.10045	0.03615
Average in opponent	0.01892	0.00222		
Colony size	37.00000	473.00000		

	P_bicolor	P_flavicornis	P_bicolor (Sum)	P_flavicornis (Sum)
Average in central	0.04339	0.40764	0.05495	0.41764
Average in opponent	0.01156	0.01000		
Colony size	111.00000	24.00000		

	P_rotoccipita	P_flavicornis	P_rotoccipita (Sum)	P_flavicornis (Sum)
Average in central	0.08115	0.07264	0.09563	0.07790
Average in opponent	0.01448	0.00525		
Colony size	61.00000	92.00000		

	P_flavicornis	P_leviuscula	P_flavicornis (Sum)	P_leviuscula (Sum)
Average in central	0.00000	0.00000	0.00000	0.00000
Average in opponent	0.00000	0.00000		
Colony size	17.00000	6.00000		

	P_flavicornis	P_rotoccipita	P_flavicornis (Sum)	P_rotoccipita (Sum)
Average in central	0.11889	0.10117	0.12222	0.10305
Average in opponent	0.00333	0.00188		
Colony size	15.00000	71.00000		

	P_rotoccipita	P_laevigata	P_rotoccipita (Sum)	P_laevigata (Sum)
Average in central	0.02421	0.09722	0.02925	0.10222
Average in opponent	0.00503	0.00500		
Colony size	53.00000	30.00000		

	P_bicolor	P_laevigata	P_bicolor (Sum)	P_laevigata (Sum)
Average in central	0.05601	0.11473	0.06093	0.12868
Average in opponent	0.00492	0.01395		
Colony size	61.00000	43.00000		

	P_furcata	P_flavicornis	P_furcata (Sum)	P_flavicornis (Sum)
Average in central	0.14896	0.00000	0.19715	0.00104
Average in opponent	0.04819	0.00104		
Colony size	720.00000	16.00000		

	P_laevigata	P_armata	P_laevigata (Sum)	P_armata (Sum)
Average in central	0.02037	0.12830	0.02116	0.20633
Average in opponent	0.00079	0.07803		
Colony size	63.00000	53.00000		

	P_armata	P_flavicornis	P_armata (Sum)	P_flavicornis (Sum)
Average in central	0.05394	0.00000	0.28424	0.00000
Average in opponent	0.23030	0.00000		
Colony size	55.00000	189.00000		

	P_laevigata	P_furcata	P_laevigata (Sum)	P_furcata (Sum)
Average in central	0.02592	0.00000	0.02936	0.00000
Average in opponent	0.00344	0.00000		
Colony size	218.00000	103.00000		

	P_flavicornis	P_armata	P_flavicornis (Sum)	P_armata (Sum)
Average in central	0.00364	0.09242	0.00364	0.22182
Average in opponent	0.00000	0.12939		
Colony size	55.00000	55.00000		

	P_flavicornis	P_laevigata	P_flavicornis (Sum)	P_laevigata (Sum)
Average in central	0.01000	0.09773	0.01048	0.11364
Average in opponent	0.00048	0.01591		
Colony size	35.00000	22.00000		

	P_rotoccipita	P_laevigata	P_rotoccipita (Sum)	P_laevigata (Sum)
Average in central	0.05720	0.02761	0.08295	0.03513
Average in opponent	0.02576	0.00752		
Colony size	44.00000	102.00000		

	P_flavicornis	P_furcata	P_flavicornis (Sum)	P_furcata (Sum)
Average in central	0.00000	0.02314	0.00000	0.02895
Average in opponent	0.00000	0.00580		
Colony size	59.00000	247.00000		

	P_flavicornis	P_laevigata	P_flavicornis (Sum)	P_laevigata (Sum)
Average in central	0.01615	0.14481	0.01615	0.15778
Average in opponent	0.00000	0.01296		
Colony size	32.00000	45.00000		

	P_laevigata	P_bicolor	P_laevigata (Sum)	P_bicolor (Sum)
Average in central	0.05567	0.04559	0.05851	0.04902
Average in opponent	0.00284	0.00343		
Colony size	47.00000	34.00000		

	P_flavicornis	P_bicolor	P_flavicornis (Sum)	P_bicolor (Sum)
Average in central	0.00000	0.11667	0.00000	0.15370
Average in opponent	0.00000	0.03704		
Colony size	3.00000	9.00000		

	P_furcata	P_laevigata	P_furcata (Sum)	P_laevigata (Sum)
Average in central	0.10803	0.00463	0.11556	0.00602
Average in opponent	0.00753	0.00139		
Colony size	301.00000	72.00000		

	P_rufipes	P_laevigata	P_rufipes (Sum)	P_laevigata (Sum)
Average in central	0.50014	0.00000	0.50883	0.00075
Average in opponent	0.00869	0.00075		
Colony size	117.00000	155.00000		

	P_furcata	P_laevigata	P_furcata (Sum)	P_laevigata (Sum)
Average in central	0.33288	0.00402	0.36622	0.00402
Average in opponent	0.03333	0.00000		
Colony size	37.00000	29.00000		

	P_furcata	P_laevigata	P_furcata (Sum)	P_laevigata (Sum)
Average in central	0.18073	0.00000	0.22649	0.00000
Average in opponent	0.04576	0.00000		
Colony size	224.00000	82.00000		

	P_thrinax	P_laevigata	P_thrinax (Sum)	P_laevigata (Sum)
Average in central	0.00000	0.00333	0.00000	0.00400
Average in opponent	0.00000	0.00067		
Colony size	46.00000	125.00000		

	P_bicolor	P_armata	P_bicolor (Sum)	P_armata (Sum)
Average in central	0.12137	0.03101	0.12308	0.06628
Average in opponent	0.00171	0.03527		
Colony size	39.00000	43.00000		

		n_total	n_wins	n_losses
Species1	P_orbihumera	3.00000	2.00000	1.00000
Species2	P_bicolor	7.00000	4.00000	3.00000
Species3	P_armata	9.00000	7.00000	2.00000
Species4	P_laevigata	16.00000	8.50000	7.50000
Species5	P_furcata	11.00000	7.00000	4.00000
Species6	P_flavicornis	18.00000	3.00000	15.00000
Species7	P_emmae	1.00000	0.00000	1.00000
Species8	P_rotoccipita	4.00000	2.00000	2.00000
Species9	P_leviuscula	1.00000	0.50000	0.50000
Species10	P_thrinax	1.00000	0.00000	1.00000
Species11	P_rufipes	1.00000	1.00000	0.00000

Note: Ties considered as 0.5 win and 0.5 loss for each colony

Colley (C) Matrix		Sp_1	Sp_2	Sp_3	Sp_4	Sp_5	Sp_6	Sp_7	Sp_8	Sp_9	Sp_10	Sp_11
	Sp_1	5.00000	-1.00000	0.00000	0.00000	0.00000	-2.00000	0.00000	0.00000	0.00000	0.00000	0.00000
	Sp_2	-1.00000	9.00000	-1.00000	-2.00000	-1.00000	-2.00000	0.00000	0.00000	0.00000	0.00000	0.00000
	Sp_3	0.00000	-1.00000	11.00000	-2.00000	-2.00000	-3.00000	-1.00000	0.00000	0.00000	0.00000	0.00000
	Sp_4	0.00000	-2.00000	-2.00000	18.00000	-4.00000	-4.00000	0.00000	-2.00000	0.00000	-1.00000	-1.00000
	Sp_5	0.00000	-1.00000	-2.00000	-4.00000	13.00000	-4.00000	0.00000	0.00000	0.00000	0.00000	0.00000
	Sp_6	-2.00000	-2.00000	-3.00000	-4.00000	-4.00000	20.00000	0.00000	-2.00000	-1.00000	0.00000	0.00000
	Sp_7	0.00000	0.00000	-1.00000	0.00000	0.00000	0.00000	3.00000	0.00000	0.00000	0.00000	0.00000
	Sp_8	0.00000	0.00000	0.00000	-2.00000	0.00000	-2.00000	0.00000	6.00000	0.00000	0.00000	0.00000
	Sp_9	0.00000	0.00000	0.00000	0.00000	0.00000	-1.00000	0.00000	0.00000	3.00000	0.00000	0.00000
	Sp_10	0.00000	0.00000	0.00000	-1.00000	0.00000	0.00000	0.00000	0.00000	0.00000	3.00000	0.00000
	Sp_11	0.00000	0.00000	0.00000	-1.00000	0.00000	0.00000	0.00000	0.00000	0.00000	0.00000	3.00000

r Matrix	
r = rank	r_1
	r_2
	r_3
	r_4
	r_5
	r_6
	r_7
	r_8
	r_9
	r_10
	r_11

b Matrix	
b = 1 + (n_w,i - n_l,i)/2	1.50000
	1.50000
	3.50000
	1.50000
	2.50000
	-5.00000
	0.50000
	1.00000
	1.00000
	0.50000
	1.50000

C*r = b	
	r_1
	r_2
	r_3
	r_4

r_5
r_6
r_7
r_8
r_9
r_10
r_11

Inverse C Matrix

0.22979	0.05061	0.03077	0.03157	0.03347	0.04918	0.01026	0.02691	0.01639	0.01052	0.01052
0.05061	0.15014	0.04734	0.05013	0.05009	0.05147	0.01578	0.03386	0.01716	0.01671	0.01671
0.03077	0.04734	0.13301	0.04996	0.05586	0.05326	0.04434	0.03441	0.01775	0.01665	0.01665
0.03157	0.05013	0.04996	0.10132	0.05929	0.05386	0.01665	0.05173	0.01795	0.03377	0.03377
0.03347	0.05009	0.05586	0.05929	0.12565	0.05863	0.01862	0.03931	0.01954	0.01976	0.01976
0.04918	0.05147	0.05326	0.05386	0.05863	0.09721	0.01775	0.05035	0.03240	0.01795	0.01795
0.01026	0.01578	0.04434	0.01665	0.01862	0.01775	0.34811	0.01147	0.00592	0.00555	0.00555
0.02691	0.03386	0.03441	0.05173	0.03931	0.05035	0.01147	0.20069	0.01678	0.01724	0.01724
0.01639	0.01716	0.01775	0.01795	0.01954	0.03240	0.00592	0.01678	0.34413	0.00598	0.00598
0.01052	0.01671	0.01665	0.03377	0.01976	0.01795	0.00555	0.01724	0.00598	0.34459	0.01126
0.01052	0.01671	0.01665	0.03377	0.01976	0.01795	0.00555	0.01724	0.00598	0.01126	0.34459

b Matrix

1.50000
1.50000
3.50000
1.50000
2.50000
-5.00000
0.50000
1.00000
1.00000
0.50000
1.50000

Solution (multiply Inverse C Matrix times b Matrix to solve for r values)

r_1	0.48294
r_2	0.44425
r_3	0.28082
r_4	0.40672
r_5	0.45064
r_6	0.12753
r_7	0.33207
r_8	0.38065
r_9	0.44764
r_10	0.37404
r_11	0.70737

Species	Body Size	Spine	Ranking
P_flavicornis	1.74400	0.45400	0.12753
P_armata	4.00000	3.45000	0.28082
P_emmae	1.70000	1.25000	0.33207
P_thrinax	2.00600	0.22300	0.37404
P_rotoccipita	1.91000	0.00000	0.38065
P_laevigata	1.84300	1.39080	0.40672
P_bicolor	1.94600	1.44100	0.44425
P_leviuscula	2.10000	0.20000	0.44764
P_furcata	1.85100	2.68400	0.45064
P_rastellata	1.88000	0.00000	0.48294
P_rufipes	2.05000	2.42000	0.70737

Table S6: GLM and GLMM results for two-species competition trials. The “Trial” variable estimates are displayed for resource discovery rate and foraging effort trials with competition trial values treated as the baseline.

**Trial**	**Variable**	**Estimate**	**Std. Error**	**z-score**	***p*-value**
P. flavicornis					
Discovery Rate	Trial	0.096	0.367	0.273	0.785
	Colony Size	0.024	0.352	5.904	**<0.001**
Worker Presence	Trial	-0.163	0.122	-1.337	0.181
	Time Bin	3.983	0.229	17.396	**<0.001**
	Colony Size	0.128	0.025	5.126	**<0.001**
Worker Number	Trial	-0.113	0.029	-3.836	**<0.001**
	Time Bin	1.581	0.054	29.507	**<0.001**
P. laevigata					
Discovery Rate	Trial	-0.864	0.284	-3.046	**0.002**
	Colony Size	-0.003	0.003	-1.011	0.312
Worker Presence	Trial	-0.837	0.110	-7.581	**<0.001**
	Time Bin	1.155	0.188	6.134	**<0.001**
	Colony Size	0.007	0.006	1.135	0.256
Worker Number	Trial	-0.045	0.015	-2.973	**0.003**
	Time Bin	0.428	0.026	16.285	**<0.001**
